# Synaptic vesicle glycoprotein 2A (SV2A) regulates kindling epileptogenesis via GABAergic neurotransmission

**DOI:** 10.1038/srep27420

**Published:** 2016-06-06

**Authors:** Kentaro Tokudome, Takahiro Okumura, Saki Shimizu, Tomoji Mashimo, Akiko Takizawa, Tadao Serikawa, Ryo Terada, Shizuka Ishihara, Naofumi Kunisawa, Masashi Sasa, Yukihiro Ohno

**Affiliations:** 1 Laboratory of Pharmacology, Osaka University of Pharmaceutical Sciences, Osaka 569-1094, Japan; 2 Institute of Laboratory Animals, Graduate School of Medicine, Kyoto University, Kyoto 606-8501, Japan; 3 Institute of Experimental Animal Sciences, Graduate School of Medicine, Osaka University, Osaka 565-0871, Japan; 4 Nagisa Clinic, Osaka 573-1183, Japan

## Abstract

Synaptic vesicle glycoprotein 2A (SV2A) is a prototype synaptic vesicle protein regulating action potential-dependent neurotransmitters release. SV2A also serves as a specific binding site for certain antiepileptics and is implicated in the treatment of epilepsy. Here, to elucidate the role of SV2A in modulating epileptogenesis, we generated a novel rat model (*Sv2a*^*L174Q*^ rat) carrying a *Sv2a*-targeted missense mutation (L174Q) and analyzed its susceptibilities to kindling development. Although animals homozygous for the *Sv2a*^*L174Q*^ mutation exhibited normal appearance and development, they are susceptible to pentylenetetrazole (PTZ) seizures. In addition, development of kindling associated with repeated PTZ treatments or focal stimulation of the amygdala was markedly facilitated by the *Sv2a*^*L174Q*^ mutation. Neurochemical studies revealed that the *Sv2a*^*L174Q*^ mutation specifically reduced depolarization-induced GABA, but not glutamate, release in the hippocampus without affecting basal release or the SV2A expression level in GABAergic neurons. In addition, the *Sv2a*^*L174Q*^ mutation selectively reduced the synaptotagmin1 (Syt1) level among the exocytosis-related proteins examined. The present results demonstrate that dysfunction of SV2A due to the *Sv2a*^*L174Q*^ mutation impairs the synaptic GABA release by reducing the Syt1 level and facilitates the kindling development, illustrating the crucial role of SV2A-GABA system in modulating kindling epileptogenesis.

Synaptic vesicle glycoprotein 2 (SV2) is a prototype protein specifically identified in the synaptic vesicles of neurons and endocrine granules^1^,^2^. SV2 consists of three isoforms, SV2A, SV2B and SV2C, which commonly possess a 12-transmembrane-spanning structure. Although SV2 was first thought to act as a vesicular transporter due to its 12-transmembrane structure, similar to the transporter proteins, it is now known that SV2 regulates exocytotic release of neurotransmitters and hormones^3^,^4^. Among SV2 isoforms, SV2A is highly expressed in the brain including the cerebral cortex, hippocampus and cerebellum^2^. Previous studies have shown that SV2A enhances action potential-dependent neurotransmitter release from the nerve terminals without altering the morphology or the number of synaptic vesicles^3^,^4^,^5^,^6^,^7^. It is also suggested that SV2A regulates the expression and trafficking the calcium sensor protein synaptotagmin (Syt) and other secretary machinary proteins^5^,^8^, and converts the synaptic vesicles into a fully Ca^2+^-responsive state during the maturation step of primed vesicles^7^. However, the precise mechanisms of SV2A in regulating synaptic release of neurotransmitters remain to be clarified.

Previous studies demonstrated that animals lacking SV2A failed to grow, exhibited severe seizures and died within 3 weeks^3^,^4^. Although the SV2A knockout hampered detailed analysis of behavioral phenotypes due to premature death of the animals, these findings imply that SV2A controls seizure induction. In addition, SV2A has been shown to bind to levetiracetam, an antiepileptic agent which is widely used to treat partial seizures, myoclonus or generalized tonic-clonic seizures in patients with epilepsy^9^,^10^,^11^,^12^. It is now known that SV2A serves as a specific binding site for the racetam derivatives including levetiracetam, brivaracetam and seletracetam. Furthermore, expressional and functional changes in SV2A have been reported in various epileptic conditions both in animals and humans^13^,^14^,^15^,^16^,^17^,^18^,^19^,^20^. Specifically, a recent study showed that a homozygous missense mutation (R383Q) in the *SV2A* gene resulted in intractable epilepsy, involuntary movements, microcephaly and developmental retardation^20^. All these findings suggest that SV2A is implicated in the pathogenesis and treatment of epileptic disorders, but detailed functions and mechanisms (e.g., neurotransmitter specificity) of SV2A in epileptogenesis remain unknown.

In order to clarify the function and mechanism of SV2A in modulating epileptogenesis, we generated *Sv2a*-targeted rats carrying a missense mutation (L174Q) in the 1^st^ transmembrane spanning region of SV2A, using gene-driven N-ethyl-N-nitrosourea (ENU) mutagenesis/MuT-POWER techniques^21^. While gross behaviors of the *Sv2a*^*L174Q*^ rats were normal, these animals exhibited a markedly high susceptibility to the development of kindling, an experimental model of epileptogenesis, and caused disrupted GABA release in the hippocampus, illustrating the crucial role of SV2A-GABA system in modulating epileptogenesis.

## Results

### Targeted mutations in the rat *Sv2a*

**Figure 1:**
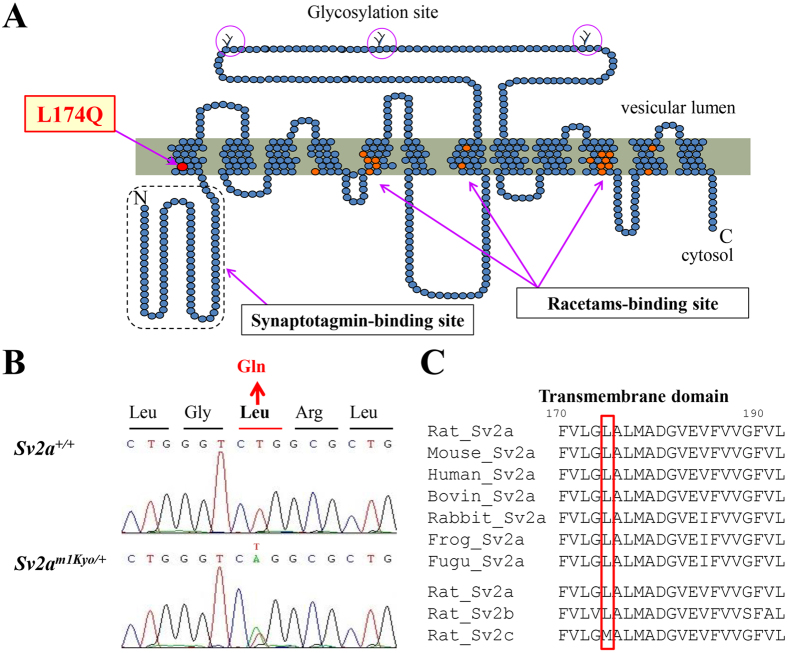
Generation of *Sv2a*^*L174Q*^ rats. (**A**) SV2A structure showing the mutation site L174Q. Glycosylation sites in a long intravesicular loop and putative binding sites for levetiracetam are also indicated. (**B**) Sequence analysis for the point mutation of the *Sv2a* gene, illustrating the presence of a single nucleotide mutation T521A. (**C**) Alignment analysis of SV2A amino acid sequences in various vertebrate species and in the rat SV2 family.

In order to generate a rat model carrying a mutation in the *Sv2a*, we used ENU mutagenesis/MuT-POWER techniques^21^, a high-throughput screening assay making use of the Mu-transposition reaction (MuT-POWER) in the Kyoto University Rat Mutant Archive (KURMA) genomic DNA library. By screening of 4608 G1 samples for mutations at the rat locus orthologous to the human *SV2A* gene, we identified a mutant *Sv2a*^*m1Kyo*^. Sequence analysis revealed that *Sv2a*^*m1Kyo*^ possesses a single nucleotide substitution, T521A, resulting in an amino acid change L174Q in the 1^st^ transmembrane spanning region which possesses a highly conserved sequence (Fig. 1). Leucine at position 174 of SV2A is identical in all vertebrates and also to the rat SV2B (Fig. 1C). A previous study using knockout-rescue techniques showed that the neighboring amino acid sequence (D179 and E182) in the 1^st^ transmembrane region is essential for the normal structure and function of SV2A^7^. Indeed, the SIFT (Sorting Intolerant From Tolerant) prediction analysis (http://sift.jcvi.org/) predicted that the L174Q substitution would be “intolerated” and markedly affect protein function.

Recovery of the identified mutant rat *Sv2a*^*L174Q*^ from frozen sperm cells was achieved by intracytoplasmic sperm injection (ICSI), yielding 10 live rats that were confirmed heterozygous for the mutations by direct sequencing (Supplementary Table 1). Heterozygous carriers were then intercrossed to produce homozygous individuals for both *Sv2a*^*L174Q*^ alleles. To eliminate mutations that may have been generated by ENU in other chromosomal regions of the *Sv2a* locus, more than 5 backcross generations (N5–N10) were performed against the F344/NSlc inbred background.

**Figure 2:**
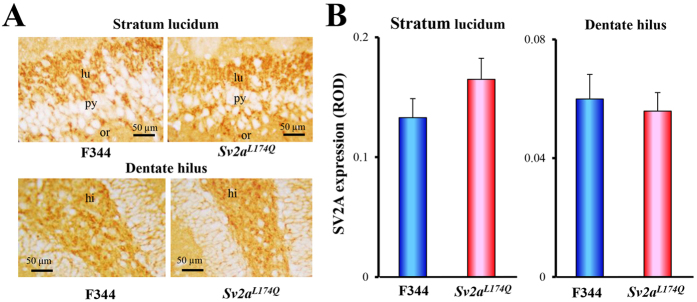
SV2A expression in *Sv2a*^*L174Q*^ rat hippocampus. (**A**) Representative photos illustrating the SV2A-immunoreactivity in the hippocampal CA3 region (stratum lucidum and dentate hilus) from *Sv2a*^*L174Q*^ and F344 rats. lu: stratum lucidum, py: pyramidal cell layer, or: stratum oriens. Hi: dentate hilus. (**B**) Immunohistochemical analysis of SV2A expression level in the striatum lucidum and dentate hilus of *Sv2a*^*L174Q*^ and F344 rats. Analyzed areas (photograph areas) are shown in Supplementary Figure. ROD: relative optical density. Each column represents the mean ± S.E.M. of 5 animals.

We first checked expressional levels and patterns of SV2A in animals homozygous for the *Sv2a*^*L174Q*^ mutation (*Sv2a*^*L174Q*^ rat) using hippocampal slices. Immunohistochemical analysis revealed that SV2A was densely expressed in the hippocampal dentate hilus and the stratum lucidum and the periphery of pyramidal neurons in the CA3 field, as reported previously^14^, and no changes in the levels and patterns of SV2A expression was observed between *Sv2a*^*L174Q*^ and F344 rats (Fig. 2).

### Susceptibility to pentylenetetrazole (PTZ)-induced seizures and kindling

**Figure 3:**
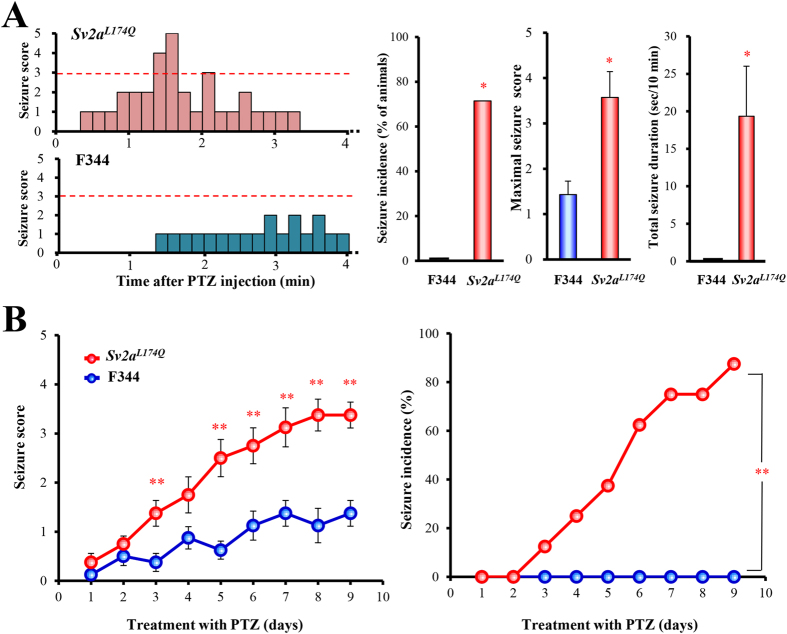
Susceptibility of *Sv2a*^*L174Q*^ rats to PTZ-induced seizures and kindling. (**A**) Left; typical time course (~4 min) of PTZ-induced excitatory behaviors and seizures evaluated by the time-sampling method (scored every 10 sec). *Sv2a*^*L174Q*^ and F344 rats were treated with an increasing dose of PTZ (10, 20 and 30 mg/kg, i.p., each 30 min interval) in a cumulative fashion. At 30 mg/kg (i.p.), PTZ induced clonic seizures in *Sv2a*^*L174Q*^ rats usually within 5 min after PTZ injection while PTZ showed no seizures in F344 rats. Right; Comparison of seizure intensity (maximal seizure score), total seizure duration and incidence rate. Each column represents the mean ± SEM or 7 rats. *p < 0.05, **p < 0.01, Significantly different from F344 rats. (**B**) Susceptibility to PTZ kindling following repeated treatments with a sub-convulsive dose (30 mg/kg/day) of PTZ. Left; Seizure intensity. Right; Seizure incidence rate (score of 3 or more). Each point represents the mean ± S.E.M. of 8 animals. **p < 0.01, Significantly different from F344 rats.

*Sv2a*^*L174Q*^ rats did not exhibit any abnormal behaviors (e.g., seizures, ataxia, motor excitation or sedation) under ordinary conditions. Either sex of *Sv2a*^*L174Q*^ rats is fertile and apparently normal in terms of development (e.g., body weight gain). However, *Sv2a*^*L174Q*^ rats showed higher susceptibility to PTZ-induced seizures than the control F344 rats (Fig. 3A). Cummulative treatments with an increasing dose of PTZ (10, 20, 30 mg/kg, i.p., each 30-min interval) evoked seizures at 30 mg/kg in *Sv2a*^*L174Q*^ rats, but not in F344 rats, yielding significantly higher seizure incidence (X^2^ = 5.00, p < 0.05), severity scores (U(12) = 7.00, p < 0.05) and duration (t(12) = 2.90, p < 0.05).

In the PTZ kindling study, *Sv2a*^*L174Q*^ and F344 rats were repeatedly treated with a sub-convulsive dose (30 mg/kg/day, i.p.) of PTZ for 9 days and, the seizures scores and incidence rates were daily monitored after the PTZ injection. As shown in Fig. 3B, *Sv2a*^*L174Q*^ rats were markedly susceptible to PTZ kindling. While the first 2 days’ treatment with PTZ showed equal seizure scores both in *Sv2a*^*L174Q*^ and F344 rats, increments in seizure scores in *Sv2a*^*L174Q*^ rats became significantly higher than in F344 rats after day 3 and reached to 3.38 ± 0.26 at day 9 (cf., 1.38 ± 0.26 in F344 rats). Incidence of PTZ seizures was also much higher in *Sv2a*^*L174Q*^ than in F344 rats (X^2^ = 11.58; p < 0.01) while none of the F344 rats showed convulsive seizures throughout the PTZ treatment period (Fig. 3B).

### Susceptibility to amygdala (AMG) kindling

**Figure 4:**
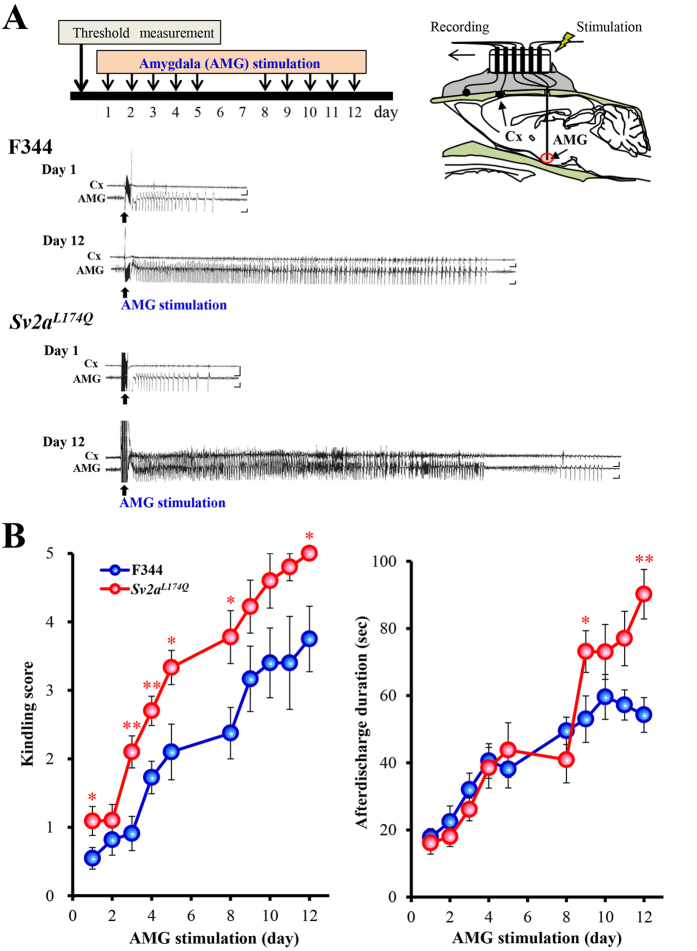
Susceptibility of *Sv2a*^*L174Q*^ rats to AMG kindling. (**A**) Representative AMG afterdischarge patterns recorded from *Sv2a*^*L174Q*^ and F344 rats following kindling stimulation (Day 1 and 12). Protocol of kindling stimulation and method of AMG afterdischarge recording are also shown in the top schema. Cx: cerebral cortex. Calibration: 1 mV, 1 s. (**B**) Susceptibility to AMG kindling following repeated AMG kindling stimulation. Left: Kindling intensity. Right: Duration of AMG afterdischarge. Each point represents the mean ± S.E.M. of 11 animals. *p < 0.05, **p < 0.01, Significantly different from F344 rats.

We next evaluated the susceptibility of *Sv2a*^*L174Q*^ rats to AMG kindling, a focal seizure model with AMG stimulation. High frequency stimulation (1 msec duration, 60 Hz for 1 sec) of AMG in conscious freely-moving rats evoked a paroxysmal afterdischarge without affecting gross behaviors (Fig. 4A). There were no differences in the afterdischarge threshold between *Sv2a*^*L174Q*^ and F344 rats (*Sv2a*^*L174Q*^ rats: 252.00 ± 45.42 μA n = 12, F344 rats: 252.08 ± 39.58 μA n = 11). However, development of AMG kindling was significantly facilitated by the L174Q mutation of *Sv2a*, illustrating faster and higher increments in the Racine’s kindling score in *Sv2a*^*L174Q*^ than in F344 rats (Fig. 4B). The duration of the AMG afterdischarges was also increased by the *Sv2a*^*L174Q*^ mutation and a significant prolongation of the afterdischarge was obtained especially in the late phase of kindling period (Fig. 4B).

### Hippocampal GABA and glutamate release

**Figure 5:**
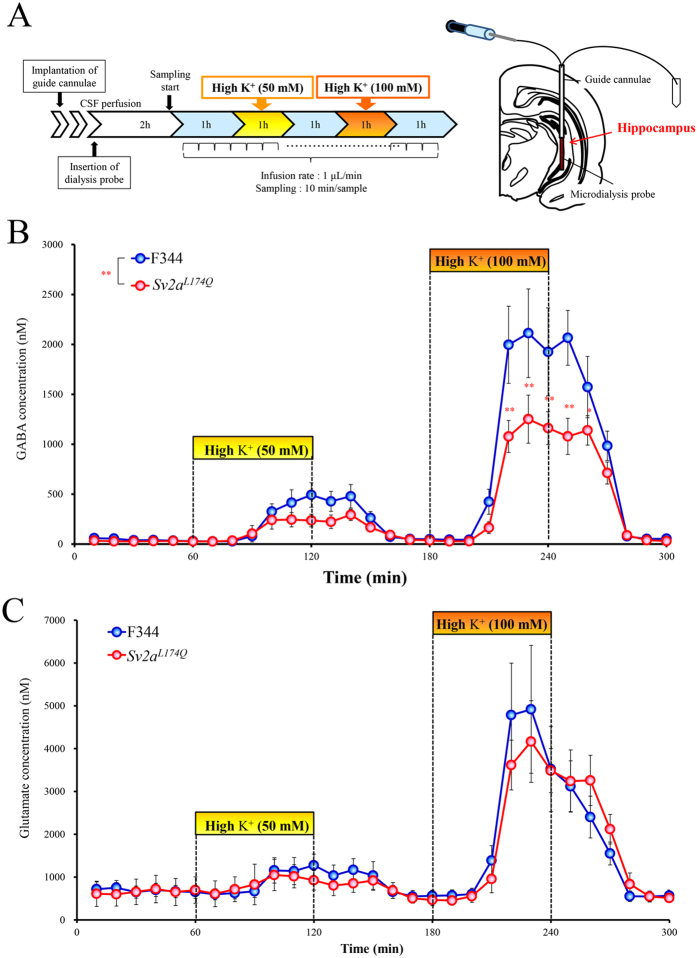
Hippocampal GABA and glutamate release. (**A**) Schematic illustrations showing the protocol of *in vivo* microdialysis experiments. Yellow and orange boxes represent the period of each depolarization stimulus with 50 and 100 mM K^+^, respectively. (**B**) Extracellular GABA level. Each point represents the mean ± S.E.M. of 6 or 10 animals. Two-way ANOVA analysis revealed statistically significant difference (*p* < 0.01) between *Sv2a*^*L174Q*^ and F344 rats. *p < 0.05, **p < 0.01, Significantly different from F344 rats. (**C**) Extracellular glutamate level. Each point represents the mean ± S.E.M. of 6 or 10 animals. There were no significant differences between *Sv2a*^*L174Q*^ and F344 rats.

Synaptic release of GABA and glutamate in the hippocampus was evaluated using *in vivo* microdialysis techniques (Fig. 5A). The basal extracellular levels of GABA or glutamate were not affected by the *Sv2a*^*L174Q*^ mutation (Fig. 5B,C). The high K^+^ (50 mM or 100 mM) stimulation concentration-dependently evoked synaptic release of GABA and glutamate both in *Sv2a*^*L174Q*^ and F344 rats. However, depolarization-induced GABA release was largely diminished by the *Sv2a*^*L174Q*^ mutation (Fig. 5B). Two-way ANOVA followed by Tukey’s test revealed that 100 mM K^+^-induced GABA release was more significantly (F(1, 404) = 20.41, p < 0.001) reduced in *Sv2a*^*L174Q*^ rats than in F344 rats. In contrast, depolarization-induced glutamate release remained unaltered (F(1, 406) = 0.33, p = 0.57) with the *Sv2a*^*L174Q*^ mutation (Fig. 5C).

**Figure 6:**
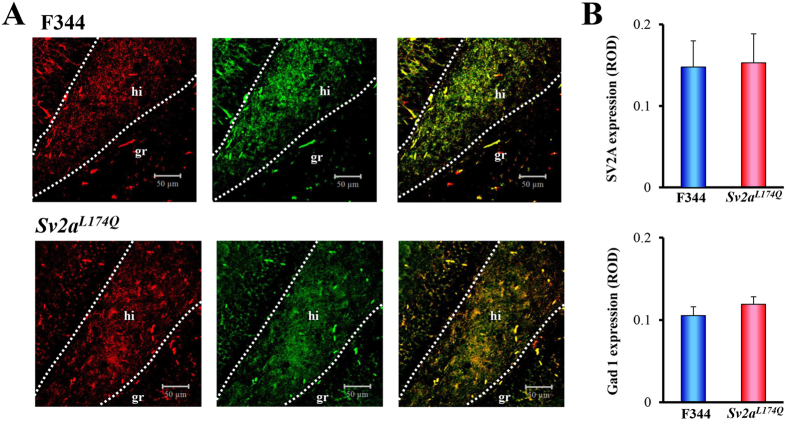
Double immunofluorescence staining of SV2A and Gad1 in the hippocampal dentate gyrus. (**A**) Representative photos illustrating double immunofluorescence staining of Gad1 (left panel: red) and SV2A (center panel: green) in the hippocampal dentate region of F344 (upper panel) and *Sv2a*^*L174Q*^ (lower panel) rats. The left panel represents a merged image of SV2A- and Gad1-immunoreactivity. hi: hilus, gr: granular cell layer. (**B**) Expressional levels of SV2A and Gad1 expression in the dentate hilus of *Sv2a*^*L174Q*^ and F344 rats. ROD: relative optical density. Each column represents the mean ± S.E.M. of 4 animals.

It should be noted that reduction of the depolarization-induced synaptic release was specific for GABAergic, but not glutamatergic, neurotransmission. To confirm the SV2A expression in GABAergic neurons, we conducted immunofluorescence double staining of SV2A and glutamate decarboxylase 1 (Gad1, a GABA-synthesizing enzyme) using a confocal laser microscope. As shown in Fig. 6A, SV2A was mostly co-stained with Gad1 in the dentate hilus (neuronal somata and dendrites) and the periphery of granular cells in the dentate gyrus (nerve terminals) both in *Sv2a*^*L174Q*^ and F344 rats. In addition, there were no significant differences between *Sv2a*^*L174Q*^ and F344 rats in the expressional level of SV2A or Gad1 (Fig. 6B).

### Expressional changes in exocytosis modulator proteins

**Figure 7:**
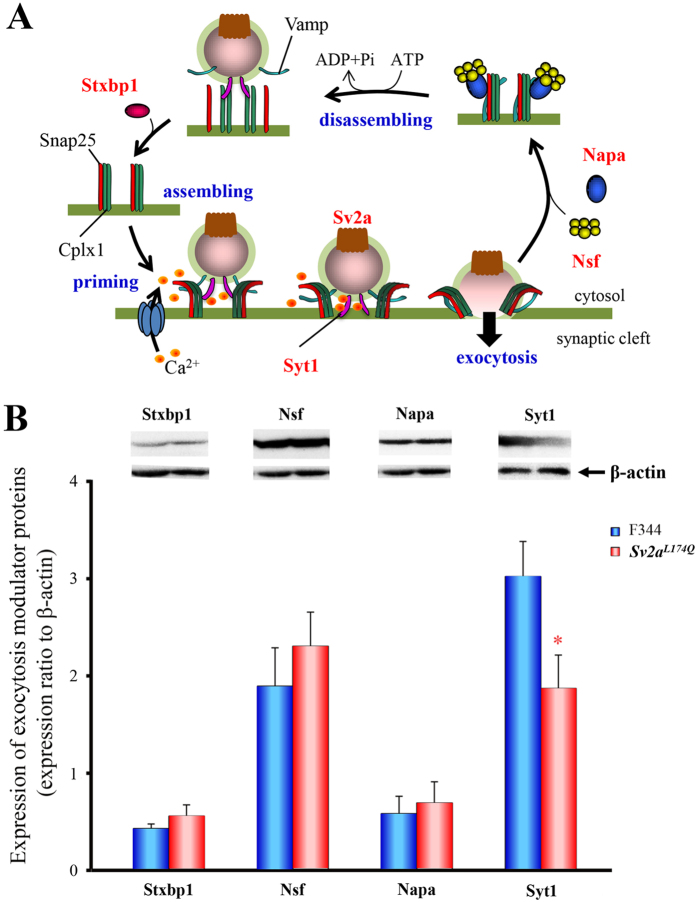
Expressional changes in exocytosis modulator proteins in *Sv2a*^*L174Q*^ rats. (**A**) Schematic illustrations showing exocytotic process and modulatory proteins. Stxbp1: syntaxin binding protein 1, Nsf: N-ethylmaleimide-sensitive factor, Napa: N-ethylmaleimide-sensitive factor alpha and Syt1. (**B**) Western blot analysis of hippocampal Stxbp1, Nsf, Napa and Syt1 in *Sv2a*^*L174Q*^ and F344 rats. Expression level of each exocytotic modulatory protein is expressed as the relative optical density ratio to β-actin. Each column represents the mean ± S.E.M. of 5 or 6 animals. *p < 0.05, Significantly different from F344 rats.

Since the L174Q mutation of *Sv2a* interrupted the depolarization-induced GABA release, we evaluated the changes in expression of exocytosis modulator proteins, syntaxin binding protein 1 (Stxbp1), N-ethylmaleimide-sensitive factor (Nsf), N-ethylmaleimide-sensitive factor alpha (Napa) and Syt1 in the hippocampus (Fig. 7A). Western blot analysis revealed that the expression of Syt1 was significantly (t(10) = 2.29, p < 0.05) decreased in *Sv2a*^*L174Q*^ rats (Fig. 7B). However, there were no significant differences in the levels of other modulator proteins, illustrating that the *Sv2a*^*L174Q*^ mutation specifically regulates the expression of Syt1, a Ca^2+^ sensor protein mediating synaptic exocytosis.

## Discussion

In the present study, we succeeded in generating a novel rat model of SV2A-related epileptic disorders, carrying the *Sv2a*^*L174Q*^ mutation. The missense mutation L174Q is located in the 1^st^ transmembrane region which contains a highly-conserved sequence among various species. A previous study using knockout-rescue techniques^7^ showed that the neighboring missense mutations of two polar amino acids (D179A and E182A) in the 1^st^ transmembrane region drastically affected the normal structure and function of SV2A. Consistent with these observations, the SIFT analysis the *Sv2a*^*L174Q*^ mutation predicted that the L174Q substitution is “intolerated” and markedly affect protein function and, indeed, it disrupted depolarization-induced GABA release.

Analysis of behavioral phenotypes revealed that the L174Q mutation of *Sv2a* gene elevated seizure sensitivity to PTZ and markedly accelerated the kindling epileptogenesis both in generalized (PTZ kindling) and focal (AMG kindling) seizure models. Previous studies showed that SV2A-knockout animals exhibited severe seizures and died within 3 weeks^3^,^4^ while heterozygous SV2A-deficient mice did not show any spontaneous seizures, but exhibited higher susceptibility to drug-induced seizures and kindling development^11^. As compared to these animals, the behavioral phenotypes of our *Sv2a*^*L174Q*^ missense model were similar to those of the SV2A-deficient mice, which was possibly due to partial loss of the SV2A function by the *Sv2a*^*L174Q*^ mutation. In addition, *in vivo* microdialysis studies revealed that the *Sv2a*^*L174Q*^ mutation preferentially inhibited depolarization-induced GABA, but not glutamate, release in the hippocampus without affecting the basal release level. Our results are consistent with a previous electrophysiological study^3^ showing that the SV2A knockout leads to a reduction in action potential-dependent IPSPs in the hippocampal CA3 region. Specific control of exocytotic GABA release by SV2A was further supported by its specific expression in GABAergic neurons and nerve terminals probed with anti-Gad1 antibody. It is therefore likely that SV2A specifically modulates inhibitory GABA release in the hippocampus, which may account for the elevated susceptibility of *Sv2a*^*L174Q*^ rats to PTZ seizures and kindling epileptogenesis.

In contrast to GABA release, depolarization-induced glutamate release in the hippocampus was not affected by the *Sv2a*^*L174Q*^ mutation. These results are consistent with our previous immunohistochemical findings in mice that SV2A was rarely co-stained with the glutamatergic marker, vesicular glutamate transporter 1, in the hippocampus, even in the stratum lucidum of the CA3 region which densely receives glutamatergic mossy fibers from the dentate gyrus and highly expresses SV2A^15^. Although an early conception that SV2A is ubiquitously present in almost all neurons, independent of their neurotransmitter type^4^, sometimes confused our understanding of SV2A function, the present study clearly illustrates the specific control of synaptic GABA release by SV2A in the hippocampus, a primary site controlling seizure induction, which may be a principal mechanism of SV2A in modulating epileptic disorders.

Although the molecular mechanisms underlying the control of synaptic release by SV2A remain to be clarified, it is suggested that SV2A regulates the expression of the Ca^2+^ sensor protein Syt1 and may change the sensitivity of synaptic vesicles to Ca^2+ ^7,8. In the present study, we found a selective down-regulation of Syt1 by the *Sv2a*^*L174Q*^ mutation among the exocytosis regulator proteins examined (i.e., Stxbp1, Nsf, Napa). Thus, it is considered likely that dysfunction of SV2A by the *Sv2a*^*L174Q*^ mutation inhibited synaptic GABA release at least partly via the down-regulation of Syt1. However, detailed mechanisms of SV2A for regulation of GABA release and/or for interaction with Syt1 are currently unknown. A previous study suggested that the neighboring missense mutation (D179A and E182A) disrupt the normal folding of SV2A and/or its trafficking to the plasma membranes^7^. Thus, further studies are required to delineate molecular mechanisms of SV2A dysfunction by the *Sv2a*^*L174Q*^ mutation.

Down-regulation or dysfunction of SV2A has been suggested to be involved in intractable TLE and focal cortical dysplasia epilepsy in humans^16^,^17^,^18^,^19^. Conversely, expression of SV2A was up-regulated by kindling stimulation, specifically in GABAergic interneurons of the hippocampal dentate hilus, possibly as a compensatory mechanism^13^,^14^,^15^. Furthermore, a recent clinical study showed that a homozygous arginine to glutamine mutation at position 383 (R383Q) in exon 5 of the *SV2A* gene resulted in intractable epilepsy and heavy developmental retardation^20^. All these findings imply that SV2A is involved in the pathogenesis of human epileptic disorders. Thus, our *Sv2a*^*L174Q*^ rats may be useful as a novel model for exploring the epileptogenic mechanisms in SV2A-related epileptic disorders. In addition, since SV2A is known to be a specific action site for levetiracetam and its derivatives (e.g., brivaracetam and seletracetam)^10^,^11^,^12^, the *Sv2a*^*L174Q*^ rat may also be useful for analyzing the actions of the racetam-derivatives and for screening or evaluating new drug candidates acting on SV2A.

In conclusion, we generated a novel *Sv2a*^*L174Q*^ rat carrying a *Sv2a*-targeted mutation (L174Q) to investigate the functional role of SV2A in modulating epileptogenesis. *Sv2a*^*L174Q*^ rats were highly susceptible to kindling by repeated PTZ treatments or focal AMG stimulation, showing faster kindling development and higher seizure severity. In addition, the *Sv2a*^*L174Q*^ mutation specifically reduced depolarization-induced GABA, but not glutamate, release in the hippocampus, which was associated with reduced expression of Syt1. The present study illustrates the crucial role of SV2A in kindling epileptogenesis via specific modulation of synaptic GABA release.

## Materials and Methods

### Animals

Male *Sv2a*^*L174Q*^ and F344/NSlc control rats (F344) were used. The animals were kept in air-conditioned rooms under a 12-h light/dark cycle and allowed *ad libitum* access to food and water. All animal experiments were approved by the Animal Research Committees of Osaka University of Pharmaceutical Sciences and Kyoto University, and were conducted according to the Committees’ regulations on animal experimentation.

### Generation of *Sv2a* ^*L174Q*^ rats

*Sv2a*^*L174Q*^ rats were generated by the gene-driven screening for a point mutation in the gene (*Sv2a*) encoding SV2A, using the MuT-POWER techniques^21^, in the KURMA. Eight independent G1 DNA samples in KURMA were pooled for subsequent PCR reactions. Primers were designed to amplify the exonic region of the rat *Sv2a* gene from approximately 50 bp flanking each intron (Supplementary Table 2). PCR reactions were carried out in a total volume of 15 μL under the following conditions: 94 °C for 3 min for 1 cycle, 94 °C for 30 sec, 60 °C for 30 sec, and 72 °C for 1 min for 35 cycles. The Mu transposition reactions were previously described^22^. The Mu transposition reaction mixture contained 33 nM labeled Mu-end DNA, 100 nM MuA transposase, PCR products, 25 mM HEPES (pH 7.6), 15% (vol/vol) glycerol, 10% DMSO, 10 mM CHAPS, 10 mM MgCl_2,_ and 300 mM NaCl. Reactions were carried out at 20 °C for 5 min and the reaction mixture was then purified by CleanSEQ (Beckman Coulter) to remove dye-labeled oligos, followed by automatic electrophoresis on a 16-capillary 3100 DNA Sequencer (Applied Biosystems). When positive peaks were detected in the 8 pooled samples by MuT-POWER screening, individual sequences of the 8 independent samples were determined. The sequencing reactions were performed with a BigDye terminator v3.1 cycle sequencing mix, followed by the standard protocol for the Applied Biosystems 3100 DNA Sequencer.

In the ICSI procedure, sperm heads were injected into denuded oocytes at ambient temperature (23 ± 2 °C) using a piezo impact-driving unit (PMM-150FU; Prime Tech) with a pulse controller (PMAS-CT150; Prime Tech). ICSI oocytes were cultured in 60 μL microdrops of mR1ECM medium at 37 °C under mineral oil in 5% CO_2_ in air. All non-degenerating one-cell oocytes and evenly cleaved two-cell oocytes at 23 to 25 h after ICSI were transferred into the oviductal ampulla of recipient Wistar/ST rats that had been previously mated with vasectomized males. Embryo transfers were performed on the day when the vaginal plug was detected (defined as Day 1). To eliminate mutations that may have been generated by ENU in other chromosomal regions of the *Sv2a* locus, more than 5 backcross generations (N5–N10) were performed against the F344/NSlc inbred background. To predict whether the amino acid substitution (L174Q) affects the SV2A protein function in *Sv2a*^*L174Q*^ rats, the SIFT prediction analysis were performed using a program at http://sift.jcvi.org/.

### SV2A immunohistochemistry

*Sv2a*^*L174Q*^ or F344 rats were decapitated under pentobarbital (80 mg/kg, i.p.) anesthesia. The brain was removed from the skull and placed in 4% paraformaldehyde solution for 24 h. After postfixation, the brain was dehydrated and embedded in paraffin. Formalin-fixed and paraffin-embedded hippocampal tissues were cut into 4 μm thick sections and immunohistochemically stained with a goat anti-rat SV2A (dilution 1:100, Santa Cruz Biotechnology) using the ABC method reported previously^14^,^25^,^26^,^27^. SV2A-immunoreactivity was visualized by the diaminobenzidine (DAB)-nickel staining. To quantify SV2A expression, digital images of each hippocampal section (one section per animal) were stored and the integrated optical density of the CA3 stratum lucidum and dentate hilus (see Supplementary Figure) was measured by computer analysis with ImageJ software (version 1.42, the National Institute of Health). In some experiments, hippocampal sections were subjected to immunofluorescence double staining with anti-SV2A and anti-Gad1^15^. Sections were incubated with a goat anti-rat SV2A (dilution 1:500, Santa Cruz Biotech) and mouse anti-human Gad1 antibody (dilution 1:1000, Santa Cruz Biotech) for 42 h at 4 °C, and then with an FITC (fluorescein isothiocyanate; green fluorescence)-conjugated rabbit anti goat IgG secondary antibody (dilution 1:500, Sigma-Aldrich, St. Louis, MO USA) and TRITC (tetramethyrhodamine-5-(and 6)-isothiocyanate; red fluorescence)-conjugated rabbit anti-mouse IgG secondary antibody (dilution 1:500, Sigma-Aldrich, St. Louis, MO USA) to probe SV2A and Gad1, respectively. Immunofluorescence images were obtained with a confocal laser scanning microscope (Carl Zeiss Japan, LSM 700 ZEN, Tokyo, Japan). To quantify SV2A and Gad1 expression, digital images of the hippocampal dentate hilus were stored and the integrated optical density was measured by computer analysis with ImageJ software (ver. 1.42, NIH).

### PTZ-induced seizures and kindling

To evaluate the acute seizure susceptibility, *Sv2a*^*L174Q*^ or F344 rats were cumulatively injected with an increasing dose of PTZ at 10, 20 or 30 mg/kg (i.p.) with a 30-min interval. Immediately after each dosing of PTZ, animals were placed in an observation cage (28 cm × 45 cm × 20 cm) and the incidence of excitation behaviors are continuously monitored by the time-sampling method (scored every 10 sec) for 10 min using a 6-point ranked scale as follows, 0: no change, 1: recurrent head twitches or wet dog shakes, 2: myoclonic jerk of forepaws and/or upper trunk, 3: marked myoclonic jerk of forepaws and/or upper trunk with a rearing posture, 4: clonic seizures, 5: marked clonic seizures with falling backward^28^,^29^,^30^. The incidence of seizures was judged as positive when the animal showed a seizure score of 3 or more, and seizure duration were also monitered. To evaluate susceptibility to PTZ kindling, a sub-convulsive dose (30 mg/kg/day, i.p.) of PTZ was given to *Sv2a*^*L174Q*^ or F344 rats for 9 days. The incidence and severity of PTZ-induced seizures were evaluated over 20 min after the daily PTZ injection using a 6-point ranked scale, as described previously.

### AMG kindling

*Sv2a*^*L174Q*^ or F344 rats were chronically implanted with recording and stimulating electrodes into the basolateral AMG (P: 2.2 mm, L: 4.8 mm and H: −7.2 mm from the cortical surface)^31^, as described previously^32^,^33^,^34^. After a recovery period (about 1 week) from the surgery, animals were placed in an electrically shielded observation cage (28 cm × 45 cm × 20 cm) and EEG was recorded under freely-moving conditions using an amplifier (MEG-6108; Nihon Kohden) and a thermal alley recorder (RTA-1100; Nihon Kohden, Tokyo, Japan). A high-frequency stimulation (1 msec duration, 60 Hz and 1 sec) was given to the AMG electrode with 50 μA increments every 5 min until an afterdischarge was evoked. The threshold was defined as the lowest intensity producing an afterdischarge (at least 5 sec duration). To evaluate the development of AMG kindling, a constant current stimulation, which was adjusted to 130% of the threshold in each animal, was given to the AMG every weekday for 2 weeks. Each day, seizure severity was scored according to Racine’s scale^35^,^36^: 1 = immobility, eye closure, twitching of vibrissae or facial clonus; 2 = head nodding with more severe facial clonus; 3 = clonus of one forelimb; 4 = clonic seizures of bilateral forelimbs with rearing; 5 = generalized clonic seizures accompanied by rearing and falling. The duration of AMG afterdischarge was also monitored.

### Hippocampal GABA and glutamate release

*Sv2a*^*L174Q*^ or F344 rats were anesthetized with pentobarbital (40 mg/kg, i.p.) and fixed in a stereotaxic instrument (Narishige, SR-6, Tokyo, Japan). A guide cannula (1 mm diameter) was inserted into a position 2 mm above the hippocampal regions (P: 5.5 mm, L: 4.6 mm, H: −4.2 mm)^31^ and fixed to the skull using dental cement. After a recovery period of about 1 week, animals with a chronically implanted guide cannula were used in microdialysis experiments. A dialysis probe (Eicom, A-I-10-02, Kyoto, Japan) was inserted into the hippocampus through a guide cannula and aCSF containing (in mM): NaCl 140, KCl 2.4, MgCl_2_ 1.0, CaCl_2_ 1.2, NaHCO_3_ 5.0, was perfused at a flow rate of 1 μL/min using a microperfusion pump (Eicom, ESP-32, Kyoto, Japan). The dialysate samples were collected in a microtube every 10 min (10 μL/sample). To evaluate the depolarization-evoked synaptic release, high K^+^ (50 or 100 mM)-containing aCSF was perfused for 60 min through the dialysis probe.

The dialysate samples were analyzed for GABA and glutamate levels using a HPLC-ECD system. GABA and glutamate were derivatized with *o*-phthaldehyde (OPA) before the HPLC injection and separated on a cation exchange colume (Eicom, 3.0φ × 150 mm; Eicompak SC-5ODS, Kyoto, Japan). The mobile phase consisted of 0.1 M phosphate buffer, 5 mg/L EDTA 2Na, pH 6.0, with 27% methanol pumped at a flow rate of 500 μL/min. All data were analyzed by using eDAQ Power Chrom (eDAQ Pty Ltd, Denistone East, Australia).

### Western blot of exocytosis modulator proteins

Brain samples were obtained from *Sv2a*^*L174Q*^ or F344 rats under pentobarbital (80 mg/kg, i.p.) anesthesia and chilled in ice-cold saline. Brains were then dissected into various region blocks and homogenized in an ice-cold lysis buffer (pH 7.5) containing: (in mM) Tris 20, NaCl 150, MgCl_2_ 10, EDTA 1.0, 1% Triton X-100 and a mixture of protease inhibitors (leupeptin, aprotinin, E-64, pepstatin A, bestatin and 4-(2-Aminoethyl) benzensulfonyl fluoride hydrochloride) (Nakarai Tesque, Tokyo, Japan). The homogenate was centrifuged at 15,000 g, 4 °C for 30 min and the supernatant was stored at −80 °C for subsequent analysis.

Western blotting was performed according to the methods described previously^14^,^23^,^24^, using the corresponding primary antibodies as follows, mouse monoclonal antibody against Stxbp1 (1:2000, Synaptic Systems), Nsf (1:2000, Synaptic Systems), Napa (1:2000, Santa Cruz Biotechnology), Syt1 (1:5000, Synaptic Systems) and β-actin (dilution 1:2000, Sigma Aldrich, St Louis, MO) and a sheep anti-mouse IgG-HRP conjugate (dilution 1:2000, GE Healthcare) as the secondary antibodies. Final detection was performed with the enhanced chemiluminescence method (Amersham ECL Western blotting detection reagents and analysis system, GE healthcare, Buckinghamshire, UK) using a lumino imaging analyzer (LAS-3000, FUJIFILM, Tokyo, Japan).

### Statistical analysis

Data are expressed as the mean ± S.E.M. Statistical significance of differences between two groups was performed by Mann-Whitney’s U-test (non-parametric) or Student’s *t*-test (parametric). Comparisons of seizure incidence rate in PTZ-induced seizure and PTZ kindling paradigm were done by the Kaplan-Meier method and compared by log-rank analysis. Differences in GABA and glutamate release (*in vivo* microdialysis) were analyzed by two-way ANOVA followed by Tukey’s post hoc multiple comparison test. A *p* value of less than 0.05 was considered statistically significant.

## Additional Information

**How to cite this article**: Tokudome, K. *et al.* Synaptic vesicle glycoprotein 2A (SV2A) regulates kindling epileptogenesis via GABAergic neurotransmission. *Sci. Rep.* **6**, 27420; doi: 10.1038/srep27420 (2016).

## Supplementary Material

Supplementary Information
