# Supplementary material for: Synaptic vesicle glycoprotein 2A (SV2A) regulates kindling epileptogenesis via GABAergic neurotransmission
Source: Sci Rep. 2016 Jun 6;6:27420. doi: 10.1038/srep27420 (PMC4893657; doi:10.1038/srep27420)
Supplement: Supplementary Information [file srep27420-s1.doc]

**Synaptic vesicle glycoprotein 2A (SV2A) regulates kindling epileptogenesis via GABAergic neurotransmission**

Kentaro Tokudome^1,+^, Takahiro Okumura^1,+^, Saki Shimizu^1^, Tomoji Mashimo^2,3^,　Akiko Takizawa ^2,†^, Tadao Serikawa^1,2^, Ryo Terada^1^, Shizuka Ishihara^1^, Naofumi Kunisawa^1^, Masashi Sasa^4^, Yukihiro Ohno^1^,*

^1^Laboratory of Pharmacology, Osaka University of Pharmaceutical Sciences, Osaka 569-1094, Japan

^2^Institute of Laboratory Animals, Graduate School of Medicine, Kyoto University, Kyoto 606-8501, Japan

^3^Institute of Experimental Animal Sciences, Graduate School of Medicine, Osaka University, Osaka 565-0871, Japan

^4^Nagisa Clinic, Osaka 573-1183, Japan

^+^K.T. and T.O. contributed equally to this work.

^†^Present address: Medical College of Wisconsin, Human Molecular Genetic Center, Milwaukee, WI 53226.

*To whom correspondence should be addressed:

Dr. Yukihiro Ohno, Laboratory of Pharmacology, Osaka University of Pharmaceutical Sciences, 4-20-1 Nasahara, Takatsuki, Osaka 569-1094, Japan.

TEL:+81-72-690-1052; FAX:+81-72-690-1053

E-mail: [yohno@gly.oups.ac.jp](mailto:yohno@gly.oups.ac.jp)

**Supplementary Tables and Figure**

| **Supplementary Table S1 Recovery of *Sv2a^L174Q^* frozen sperm by ICSI** | | | |
| --- | --- | --- | --- |
| Injected Oocytes | Transferred Oocytes | Born (%) | Mutated |
| 203 | 156 (76.8%) | 17 (8.4) | 10 (♂4, ♀6) |

| **Supplementary Table S2 List of primers used for MuT-POWER screening** | | | |
| --- | --- | --- | --- |
| Name | Fowerd | Reverse | Product (bp) |
| Sv2a_exon2 | ATTGAGCAAAGGGCTGAAAA | ATGGTGTTTGGGGAGTCAGT | 790 |
| Sv2a_exon3 | ACCCCCATTTCCTCCTCTC | AGGGCACAGTTCTGCCTTTA | 236 |
| Sv2a_exon4.5 | GGGAAGATGGCTGTTTCCTA | AAGAAGCGGTCAGGAGAGTG | 491 |
| Sv2a_exon6 | GAAGCCAGACTGAAGGGATG | ATCTAGGAAATGGGCGACCT | 246 |
| Sv2a_exon7.8 | TTGGAATGGGTTGGGAAGTA | CTTCAGCCACACACAACCAC | 456 |
| Sv2a_exon9 | TGTCTCATCTGCCCTGTCTG | CCCCCAACACACACACAC | 250 |
| Sv2a_exon10 | GACTAAGGGGGCATGTCTCA | CTCCTCCTCCCTTCATCACA | 223 |
| Sv2a_exon11 | GGTAGCCGTTCTCATGTTCG | CCAGGCTGCCTCTGTTCC | 299 |
| Sv2a_exon12 | TCACGGCTGTCTCTTCTCCT | CCAGTCCTGTAGCTCCCAAA | 242 |
| Sv2a_exon13 | ACCTGCCCTTCTCCCTTC | CATCCCTAAAGCCCCTGAG | 275 |

**Supplementary Figure S1 Schema showing the areas (i.e., CA3 stratum lucidum and dentate hilus) analyzed for SV2A expression (also photographed) in the hippocampus**


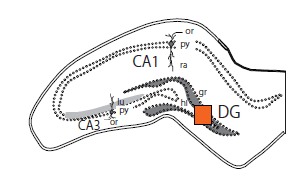


Stratum lucidum

Dentate hilus
